# Comparative Analysis of Open, Laparoscopic, and Robotic Pancreaticoduodenectomy: A Systematic Review of Randomized Controlled Trials

**DOI:** 10.3390/medicina61071121

**Published:** 2025-06-21

**Authors:** Valentina Valle, Paraskevas Pakataridis, Tiziana Marchese, Cecilia Ferrari, Filippos Chelmis, Iliana N. Sorotou, Maria-Anna Gianniou, Aleksandra Dimova, Oleg Tcholakov, Benedetto Ielpo

**Affiliations:** 1 Division of General, Minimally Invasive and Robotic Surgery, Department of Surgery, University of Illinois at Chicago, Chicago, IL 60607, USA; 2 Faculty of Medicine, Universitat Pompeu Fabra (UPF), 08003 Barcelona, Spain; 3 University Hospital Lozenetz, 1407 Sofia, Bulgaria; pakataridisparaskevas@gmail.com (P.P.);; 4 Lecce General and HPB Surgery Department, Vito Fazzi Hospital, 73100 Lecce, Italy; 5 Department of General and Vascular Surgery, Upper GI and HPB Department, Tallaght University Hospital, Trinity College, D02 PN40 Dublin, Ireland; 6 Division of Hepato-Biliary-Pancreatic Surgery, Department of Surgery, Hospital de la Santa Creu i Sant Pau, Universitat Autonoma de Barcelona, 08193 Barcelona, Spain; 7 Faculty of Medicine, Sofia University “St. Kliment Ohridski”, 1504 Sofia, Bulgaria; philipposchelmis8@gmail.com (F.C.); isorotou16@gmail.com (I.N.S.);; 8 Hpb Unit, Hospital Del Mar, 08003 Barcelona, Spain

**Keywords:** pancreatic cancer, pancreaticoduodenectomy, open pancreaticoduodenectomy, robotic pancreaticoduodenectomy, laparoscopic pancreaticoduodenectomy, randomized controlled trials, systematic review

## Abstract

*Background and Objectives*: Various publications have compared outcomes among open (OPDs), laparoscopic (LPDs), and robotic pancreaticoduodenectomies (RPDs); however, the number of randomized controlled trials (RCTs) remains limited. This study aims to conduct a systematic review and analyze the outcomes between these approaches from randomized controlled trials. *Materials and Methods*: We performed a systematic literature search across PubMed/MedLine, Cochrane Library, ClinicalTrials.gov, and Google Scholar to identify relevant RCTs. The systematic review was conducted using the reporting items for systematic reviews and network meta-analyses guidelines (PRISMA-NMA) and registered in Prospero (CRD420251024475). For statistical analysis R software (version 4.3.2) was used. *Results*: Eight RCTs involving 1416 patients (706 OPDs, 600 LPDs, 110 RPDs) were included. LPD had a significantly longer operative time than OPD, while RPD showed no significant difference compared to OPD. Blood loss was reduced in both minimally invasive approaches. LPD showed a higher R0 resection rate and lower pancreatic fistula rate, whereas RPD had the lowest mortality. No significant differences were observed in major complications, reoperation, or readmission. LPD shortened hospital stay; RPD showed no difference. *Conclusions*: Although open pancreaticoduodenectomy remains a well-established standard, both laparoscopic and robotic approaches offer safe alternatives with distinct advantages. LPD is associated with shorter hospital stay and lower pancreatic fistula rates, whereas RPD demonstrates the lowest mortality. The lack of direct randomized comparisons between LPD and RPD highlights the need for further head-to-head trials.

## 1. Introduction

Pancreatic and periampullary tumors represent a heterogeneous group of malignancies, with pancreatic ductal adenocarcinoma (PDAC) being the most common and aggressive histologic subtype. Pancreatic cancer remains the seventh leading cause of cancer-related mortality worldwide, with a five-year overall survival rate of less than 10%, largely due to late presentation and limited efficacy of systemic therapies [1,2]. Tumors located in the pancreatic head, distal bile duct, ampulla of Vater, or duodenum often present earlier due to biliary obstruction, making them more amenable to curative-intent surgery [3]. However, even among resectable tumors, recurrence rates remain high, with margin-negative (R0) resection and adequate lymphadenectomy being critical prognostic factors [4]. Multimodal management—including neoadjuvant or adjuvant chemotherapy—is increasingly integrated into treatment algorithms, particularly for borderline-resectable or high-risk patients [5]. The complexity of surgical resection in this region is compounded by the proximity to major vascular structures and the requirement for precise dissection and reconstruction. Therefore, pancreaticoduodenectomy (PD) remains a cornerstone in the management of these malignancies and continues to evolve in pursuit of improved outcomes.

PD is a complex surgical resection that offers the only potentially curative treatment for malignant tumors of the pancreatic head, distal bile duct, and ampullary region, as well as selected benign periampullary lesions. Over recent decades, advancements in surgical technique and perioperative care—along with centralization of pancreatic surgery to high-volume centers—have substantially reduced operative mortality in specialized units [6]. Nevertheless, PD continues to be associated with significant morbidity, with postoperative complication rates approaching 40% even in expert centers. This persistent morbidity has motivated efforts to further improve outcomes through minimally invasive surgery [7]. The evolution from open pancreaticoduodenectomy (OPD) to minimally invasive approaches began in the 1990s. Gagner and Pomp in 1994 reported the first laparoscopic PD (LPD), demonstrating the feasibility of a fully laparoscopic Whipple in a small series [8]. Since then, improvements in laparoscopic instrumentation and surgeon experience have gradually expanded the use of LPD. Nonrandomized studies from high-volume centers have suggested that LPD could achieve postoperative outcomes comparable to OPD [9]. However, the steep learning curve and technical challenges of LPD—requiring advanced skills in laparoscopic dissection and reconstruction—have raised concerns about increased morbidity and mortality when performed outside of specialized teams [10]. These issues came to the forefront with the multicenter LEOPARD-2 trial in Europe, which was stopped prematurely after LPD was associated with higher 90-day mortality [11].

In parallel, the robotic platform emerged in the early 2000s as an alternative minimally invasive approach for PD. Robotic PD (RPD), first introduced by Giulianotti et al. in 2003, aimed to overcome the limitations of laparoscopy by offering enhanced three-dimensional visualization and wristed instrument articulation [12]. Advocates of RPD argue that it may shorten the learning curve for minimally invasive PD and facilitate complex maneuvers during the reconstruction phase [13]. Over the past decade, many high-volume centers have adopted RPD, and nonrandomized comparisons suggested that this approach could achieve similar or better outcomes to OPD, with some series noting reduced blood loss and shorter hospital stays.

This study aims to evaluate the current evidence from randomized controlled trials comparing the outcomes of open, laparoscopic, and robotic approaches to pancreaticoduodenectomy.

## 2. Materials and Methods

### 2.1. Registration and Reporting

The Preferred Reporting Items for Systematic Reviews and Meta-Analyses 2020 guidelines were followed when reporting this systematic review [14]. The systematic review was registered in the International Prospective Register of Systematic Reviews (PROSPERO) (CRD420251024475).

### 2.2. Search Strategy

Two authors (V.V. and P.P.) independently conducted a systematic literature search. Four electronic databases (PubMed/MedLine, Cochrane Library, ClinicalTrials.gov, and Google Scholar) were searched throughout January 2025. RCTs comparing OPD to LPD, OPD to RPD, and LPD to RPD were eligible. A snowballing approach was used by screening the reference list of the articles found in the initial search. Additional potentially eligible articles were searched by the PubMed function “related articles.” The clinical trials registry (www.clinicaltrials.gov) was searched for ongoing clinical trials; however, preliminary results of ongoing trials were not included in the analysis. Duplicates and conference abstracts without accessible full texts were excluded. The remaining articles were filtered in a stepwise manner. Two authors (V.V. and P.P.) reviewed the full text of the articles to confirm their eligibility for inclusion, and the lists of eligible articles were cross-checked between the authors. The process of literature review and article selection was under the supervision of the senior author (O.T.).

### 2.3. Search Keywords

The following keywords were used in the database search: “pancreatic cancer”, “pancreaticoduodenectomy”, “open pancreaticoduodenectomy”, “robotic pancreaticoduodenectomy”, “laparoscopic pancreaticoduodenectomy”, “randomized controlled trial”, and “outcomes”. The following Medical Subject Headings terms were used in the search process: (Adult), (Laparoscopy), (Pancreaticoduodenectomy), (Randomized Controlled Trials), (Robotic Surgical Procedures), and (Open Surgical Procedures), combined using appropriate Boolean operators.

### 2.4. Article Selection Criteria

We included RCTs that compared the outcomes of OPD, LPD, and RPD. Studies were excluded if they were nonrandomized cohort studies, case reports, case series, editorials, previous reviews, or meta-analyses. There were no restrictions regarding the article language, sample size, or follow-up duration. In cases where the same cohort of patients was published twice, the most recent and larger dataset was selected for inclusion. For the eligibility of studies, the PICO criteria were used. The population included adult patients (≥18 years) undergoing elective pancreaticoduodenectomy for malignant, premalignant, or benign tumors of the pancreatic head or periampullary region. The interventions examined were OPD, LPD, or RPD. Comparators consisted of direct or indirect comparisons between at least two of these surgical techniques. Only RCTs were included to ensure high-quality evidence. The primary outcomes assessed were major postoperative complications (Clavien–Dindo grade ≥ III), 90-day mortality, and length of hospital stay. Secondary outcomes included operative time, blood loss, R0 resection rate, lymph node yield, pancreatic fistula, delayed gastric emptying, post-pancreatectomy hemorrhage, and readmission rates.

### 2.5. Assessment of Risk of Bias

The risk of bias in each included randomized controlled trial was independently evaluated by two reviewers (V.V. and P.P.) using the NIH Study Quality Assessment Tool for Controlled Intervention Studies. Each study was rated as Good, Fair, or Poor based on internal validity. Discrepancies in assessment were resolved by consensus.

### 2.6. Data Extraction

Data were independently extracted by two authors using a standardized Excel (Microsoft Corp. One Microsoft Way, Redmond, WA 98052-6399, USA.) spreadsheet. The following information was collected from each included study: first author and country of origin; total number of patients and allocation per surgical group (OPD, LPD, RPD); and baseline characteristics including age, sex, and body mass index (BMI). Operative variables included mean operative time and conversion to open surgery (if applicable). Postoperative outcomes encompassed overall complications, major complications (defined as Clavien–Dindo grade > II), 90-day postoperative mortality, and clinically relevant pancreatic fistula. Additional data were extracted on delayed gastric emptying, post-pancreatectomy hemorrhage, length of hospital stay, and readmission within 30 days. Oncologic and surgical quality metrics included R0 resection rate, lymph node yield, and duration of follow-up (in months).

### 2.7. Statistical Analysis

A pairwise meta-analysis was performed using R software (version 4.4.2) (packages: meta, netmeta, and dplyr) and Python (version 3.8.1) (packages: pandas, numpy, and matplotlib. Effect sizes were calculated using odds ratios (ORs) with 95% confidence intervals (CIs). Statistical heterogeneity was evaluated using the Cochrane Q test (*p*-value) and I^2^ statistics, with I^2^ <25% considered low, 25–75% moderate, and >75% high. A fixed-effects model was used when heterogeneity was negligible; otherwise, a random-effects model was applied. For the network meta-analysis, a Bayesian hierarchical model was employed to allow both direct and indirect comparisons among OPD, LPD, and RPD.

## 3. Results

### 3.1. Study and Patients’ Characteristics

**Figure 1:**
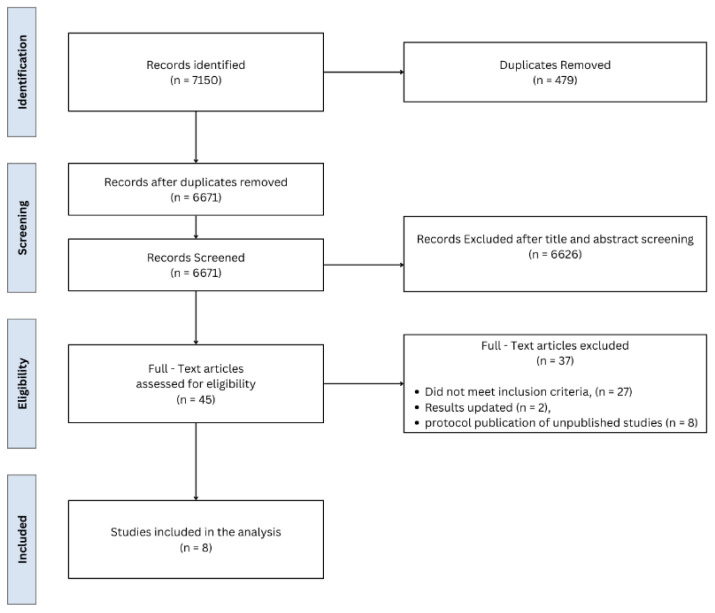
Preferred Reporting Items for Systematic Reviews and Meta-Analyses flow chart for study selection.

After screening the records of 7150 studies, the 8 RCTs that met the inclusion criteria were included in the analysis, as depicted in the PRISMA flow diagram. The studies were published between 2017 and 2024 and were conducted in a variety of countries: one in India, one in Spain, one in the Netherlands, three in China, one in South Korea, and one in Germany (NCT0281131, ISRCTN93168938, NTR5689, NCT03785743, ChiCTR2200056809, NCT03138213, DRKS00020407, and NCT03870698) [Figure 1] [11,15,16,17,18,19,20,21].

A total of 1416 patients were included in the selected studies: 706 underwent OPD, 600 underwent LPD, and 110 underwent RPD. The mean age was 62.01 years in the OPD group, 62.90 in the LPD group, and 63.35 in the RPD group. A total of 833 (58.8%) were male and 595 (41.2%) were female [Table 1].

### 3.2. Quality Assessment and Certainty of Evidence

**Figure 2:**
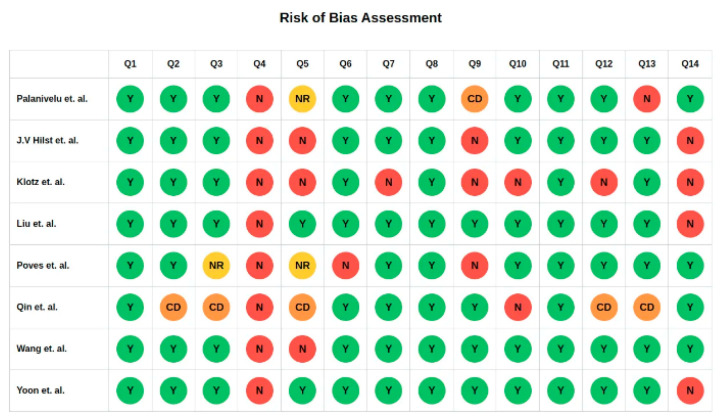
National Institute of Health Quality Assessment of Controlled Intervention Studies. Adapted from: Palanivelu et al. (2017) [15], Hilst et al. (2019) [11], Klotz et al. (2024) [21], Liu et al. (2024) [20], Poves et al. (2018) [16], Qin et al. (2024) [18], Wang et al. (2023) [17], Yoon et al. (2024) [19].

Methodological quality was assessed using the NIH Quality Assessment Tool for Controlled Intervention Studies. The overall risk of bias was considered moderate, with no study excluded due to high risk of bias. Details are summarized in Figure 2.

### 3.3. Operative Outcomes

**Figure 3:**
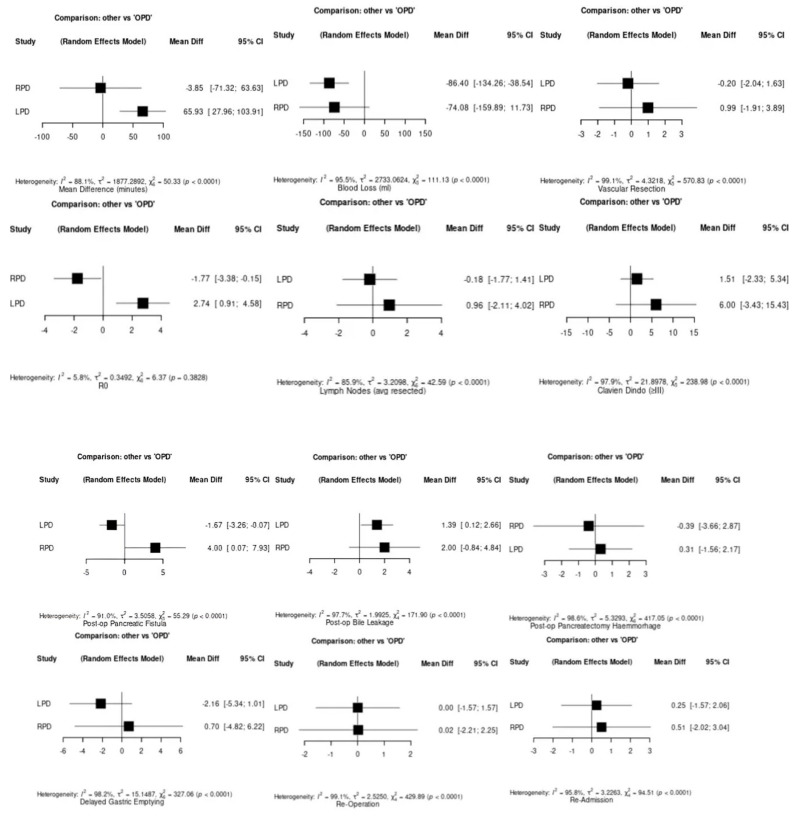

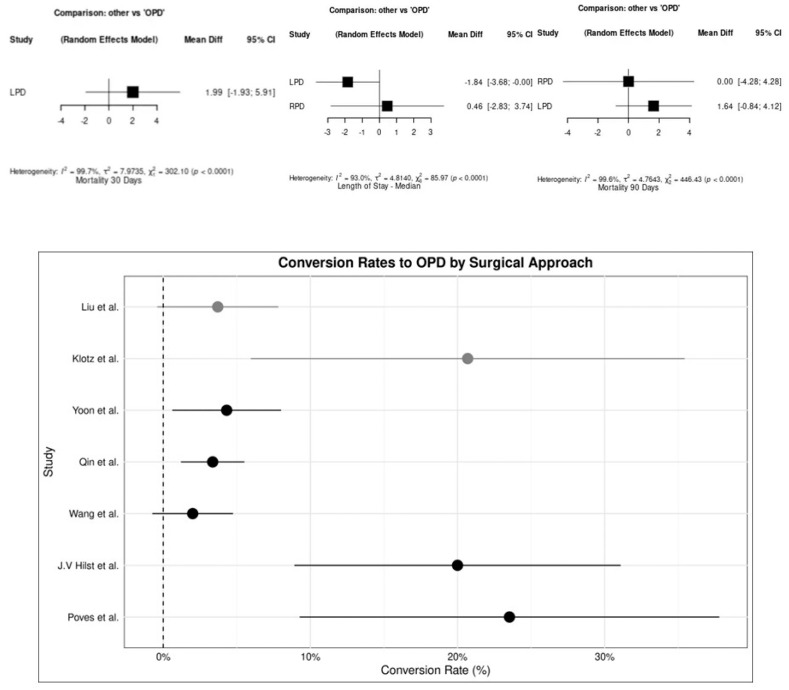
Forest plots of pairwise and network meta-analysis comparing outcomes of OPD, LPD, and RPD. Outcomes include operative time, blood loss, vascular resection, R0 resection rates, lymph node yield, Clavien–Dindo ≥ III complications, postoperative pancreatic fistula, bile leakage, hemorrhage, delayed gastric emptying, reoperation, readmission, 30- and 90-day mortality, and length of stay. Bottom right: conversion rates to open surgery from LPD and RPD. Values are presented as mean differences with 95% confidence intervals. Adapted from: Palanivelu et al. (2017) [15], Hilst et al. (2019) [11], Klotz et al. (2024) [21], Liu et al. (2024) [20], Poves et al. (2018) [16], Qin et al. (2024) [18], Wang et al. (2023) [17], Yoon et al. (2024) [19]. The black cubes represent the point estimates of each comparison, with their size indicating the weight of the data in the meta-analysis. Horizontal lines show 95% confidence intervals.

Operative time was significantly longer for LPD compared to OPD (mean difference [MD]: 59.4 min, 95% CI: 24.0 to 94.8, *p* = 0.001). In contrast, no significant difference was observed between RPD and OPD (MD: 2.18 min, 95% CI: −112.29 to 116.65, *p* = 0.97). Network meta-analysis confirmed that LPD had significantly increased operative time (MD: 65.93 min, 95% CI: 27.96 to 103.91, *p* < 0.0001), while RPD did not differ significantly (MD: −3.85 min, 95% CI: −71.32 to 63.63). Both minimally invasive techniques demonstrated reduced blood loss compared to OPD. LPD showed a significant reduction (MD: −86.40 mL, 95% CI: −134.26 to −38.54), whereas RPD showed reduction without statistical significance (MD: –74.08 mL, 95% CI: −159.89 to 11.73). No significant differences were observed in the frequency of vascular resections. LPD was associated with a significantly higher R0 resection rate (MD: 2.74%, 95% CI: 0.91 to 4.58), whereas RPD showed a small but significant decrease (MD: −1.77%, 95% CI: −3.38 to −0.15). Lymph node yield was similar across all approaches. LPD showed no significant difference from OPD in major complications (MD: 1.51%, 95% CI: −2.33 to 5.34), while RPD showed a higher rate without significance (MD: 6.00%, 95% CI: −3.43 to 15.43) Across 16 study arms, the mean number of Clavien–Dindo grade ≥ III complications was 21.6 (SD 19.88), ranging from 3 to 76 events. When comparing to OPD, the pooled mean difference for LPD was 1.51 (95% CI: −2.33 to 5.34), indicating no statistically significant difference. In contrast, RPD demonstrated a non-significant higher rate of major complications with a mean difference of 6.00 (95% CI: 3.43 to 15.43). Heterogeneity across studies was substantial (I^2^ = 97.9%, *p* < 0.0001), reflecting inter-study variability in patient selection, complication reporting, and surgical expertise. LPD was associated with a significantly lower rate of postoperative pancreatic fistula (MD: −1.67%, 95% CI: −3.26 to −0.07, *p* > 0.05), while RPD was associated with a significantly higher rate (MD: 4.00%, 95% CI: 0.07 to 7.93). These findings highlight opposing trends between the two minimally invasive techniques. LPD was associated with a statistically significant increase in bile leakage (MD: 1.39%, 95% CI: 0.12 to 2.66), while RPD showed a non-significant increase (MD: 2.00%, 95% CI: −0.84 to 4.84). No significant differences in post-pancreatectomy hemorrhage were found for LPD (MD: 0.31%, 95% CI: −1.56 to 2.17) or RPD (MD: −0.39%, 95% CI: −3.66 to 2.87). Furthermore, LPD showed a non-significant reduction in delayed gastric emptying (MD: −2.16%, 95% CI: −5.34 to 1.01), while RPD showed a non-significant increase (MD: 0.70%, 95% CI: −4.82 to 6.22). No statistically significant differences were found in reoperation (LPD: MD 0.00%, 95% CI: −1.57 to 1.57; RPD: MD 0.02%, 95% CI: −2.21 to 2.25) or readmission rates (LPD: MD 0.25%, 95% CI: −1.57 to 2.06; RPD: MD 0.51%, 95% CI: −2.02 to 3.04). Additionally, LPD was associated with no significant increase in 30-day mortality (MD: 1.99%, 95% CI: −1.93 to 5.91). No deaths were reported in the RPD group. Similarly, 90-day mortality did not differ significantly among groups (LPD: MD 1.64%, 95% CI: −0.84 to 4.12; RPD: MD 0.00%, 95% CI: −4.28 to 4.28), with high heterogeneity for both outcomes. LPD was associated with a significantly shorter hospital stay (MD: −1.84 days, 95% CI: −3.68 to −0.00), while RPD showed no difference (MD: 0.46 days, 95% CI: −2.83 to 3.74). LPD had a mean conversion rate of 10.6% (median: 4.3%, range: 2.0% to 23.5%) and RPD had a mean of 12.2% (median: 12.2%, range: 3.7% to 20.7%). Variability was considerable across studies and may reflect differences in experience, technical complexity, and patient selection [Figure 3].

## 4. Discussion

PD, commonly referred to as the Whipple procedure, has undergone substantial evolution since its first successful execution by Allen Whipple in the 1930s. Initially associated with very high morbidity and mortality, PD has become a routine operation, particularly in high-volume hepatopancreatobiliary centers [22,23]. The growing incidence of pancreatic and periampullary tumors, especially in aging populations, has increased the need for operative treatment, and thus high-quality evidence is required to ensure proper patient selection and type of operative management [24]. Simultaneously, the recognition of centralization for improved outcomes has led to the further development of pancreatic surgery specialized units, where multidisciplinary care, high-volume, and standardized postoperative pathways can lead to better outcomes [25]. Advances in cross-sectional imaging and endoscopic ultrasonography have facilitated earlier diagnosis and better preoperative staging, improving patient selection and surgical planning [26]. As pancreatic cancer continues to have high mortality, surgical resection intervention is required for most cases, emphasizing the importance of continuous refinement in operative techniques and perioperative management.

The optimal surgical approach for pancreaticoduodenectomy remains a topic of considerable interest and debate within the surgical community. While minimally invasive procedures have increased in the last decade, due to potential benefits like reduced hospital stay and faster recovery, evidence from the recent RCTs, comparing the outcomes against LPD, RPD, and the open approach is essential.

Six RCTs have compared LPD to the conventional open approach. Palanivelu et al. first demonstrated that LPD was associated with reduced blood loss and shorter hospital stay, despite longer operative times [15]. Similarly, Poves et al. observed a significant reduction in severe complications and hospital stay in the LPD group, with prolonged operative times [16]. The LEOPARD-2 trial raised concerns due to higher 90-day mortality in the LPD group, terminating the trial prematurely. The analysis performed in this study, utilizing a broader dataset, did not replicate these results, suggesting that outcomes may vary significantly depending on surgeon experience, center volume, and institutional learning curves [11]. Wang et al. reported equivalent short-term outcomes between LPD and OPD in a cohort of pancreatic cancer patients [17]. Furthermore, Qin et al. demonstrated the long-term oncologic noninferiority of LPD and its role in facilitating earlier starting of adjuvant chemotherapy, which could lead to improved long-term outcomes [18]. Most recently, Yoon et al. found that LPD accelerated functional recovery without compromising surgical safety, further supporting its use in selected patients in high-volume centers [19].

Two RCTs to date have directly compared RPD to OPD, providing insights into the safety and efficacy of RPD. Liu et al. conducted a multicenter trial across high-volume centers in China and demonstrated that RPD was associated with a significantly shorter hospital stay (median 11 vs. 13.5 days, *p* = 0.029) without increasing the incidence of severe complications, reoperations, or mortality [20]. Similarly, the EUROPA trial reported comparable overall postoperative morbidity between RPD and OPD, with absence of mortality in the RPD group and three deaths occurring in the OPD arm [21]. However, they reported increased rates of delayed gastric emptying (34.4% vs. 6.0%) and pancreas-specific complications in the RPD group but these findings did not lead to higher overall morbidity or reoperation rates. These findings are in line with this study, which showed that RPD was associated with the lowest observed mortality among all surgical modalities, and with comparable rates of hemorrhage, bile leak, and readmission to OPD. Notably, while our study showed no significant difference in operative time between RPD and OPD, it did highlight a higher rate of postoperative pancreatic fistula in the RPD group. Additionally, RPD showed a trend toward lower intraoperative blood loss and no difference in length of stay. Notably, RPD had a higher average conversion rate (12.2%) compared to LPD (10.6%), reflecting possible challenges with dissection in difficult planes or surgeon experience during the learning curve. A recent retrospective study by Chen et al. evaluated outcomes between robotic and laparoscopic pancreaticoduodenectomy using propensity score matching and learning curve analysis. The study demonstrated that RPD was associated with significantly lower rates of conversion to open surgery, reduced intraoperative blood loss, higher rates of R0 resection, and improved postoperative pancreatic fistula outcomes compared to LPD [27]. The findings support that RPD, when performed in experienced centers, is a possible alternative to open surgery with a comparable morbidity.

Despite the growing implementation of both laparoscopic and robotic techniques for pancreaticoduodenectomy, no RCTs have been published comparing LPD to RPD directly. In our comprehensive systematic search, we were unable to identify any completed or published RCTs making this direct comparison. Only one such trial was found to be registered on clinical trial databases, but it remains in a “not yet recruiting” status, noting the gap in prospective comparative evidence [28]. The available literature consists largely of comparisons through retrospective cohort studies and reviews. Without high-level direct evidence, definitive conclusions about the relative superiority of RPD versus LPD remain speculative. The IDEAL Collaboration and other surgical evaluation frameworks have showcased the need for rigorous, prospective trials to inform clinical decision-making in complex minimally invasive surgery [29].

Several retrospective studies and meta-analyses have attempted to elucidate the differences between the techniques. Zureikat et al., in a large multicenter study, reported that RPD was associated with significantly lower conversion rates and improved lymph node yield compared to LPD, with comparable morbidity and mortality profiles [30]. A meta-analysis by Nickel et al. also found that RPD was associated with reduced estimated blood loss, shorter hospital stays, and a higher likelihood of achieving R0 resection [31]. However, the higher cost and longer operative time often reported with RPD, particularly during the learning curve, are important limitations to be considered.

The findings align with the recently published studies which also compared OPD, LPD, and RPD using either observational or randomized data. Kabir et al. included 4 RCTs and 23 propensity-score matched studies (n = 4945) and found that RPD resulted in significantly less blood loss than both LPD and OPD, with both RPD and LPD showing reduction in delayed gastric emptying and wound infections compared to OPD, and shorter hospital stays for LPD in particular [32]. Aiolfi et al. analyzed 41 studies (n = 56,440), noting that both LPD and RPD were associated with reduced estimated blood loss, pulmonary and infectious complications, and length of stay, while having no differences in R0 resection or lymph node yield across approaches [33]. Joseph et al. (n = 1336) reported significantly shorter hospital stays and lower blood loss with LPD compared to OPD, although operative time was significantly increased [34]. Compared to these prior analyses, our study incorporates additional contemporary RCT data and provides an updated wide range of perioperative and oncologic outcomes.

In parallel with the evolution of surgical techniques, perioperative management strategies have advanced significantly to enhance outcomes following pancreaticoduodenectomy. The implementation of Enhanced Recovery After Surgery (ERAS) protocols has been associated with reduced rates of delayed gastric emptying, fewer pulmonary complications, and shorter lengths of stay, without increasing morbidity or readmission [35,36]. A growing body of evidence supports that adherence to ERAS principles, especially early mobilization, opioid-sparing analgesia, and standardized fluid management, can be beneficial in minimally invasive approaches, although more evidence is required for PD [37]. In addition, the integration of intraoperative technologies such as indocyanine green (ICG) fluorescence imaging has shown promise in real-time assessment of tissue perfusion, potentially mitigating the risk of ischemia-related complications including pancreatic fistula and anastomotic failure [38,39].

Patient selection is incorporating patient frailty indices, sarcopenia status, and cardiopulmonary reserve. Meta-analyses have demonstrated that frailty is a significant predictor of postoperative morbidity and mortality, regardless of surgical approach [40,41]. The development of prehabilitation programs aimed at improving nutritional and functional status prior to surgery has been developing in the recent decades, and could further assist in a reduction in perioperative morbidity and mortality [42]. The safe implementation of LPD and RPD requires a structured institutional framework, including mentorship models, case volume thresholds, and standardized criteria. Studies have shown that high-volume centers with dedicated minimally invasive HPB teams achieve superior outcomes, underscoring the importance of centralization and specialized training pathways [30,43].

Beyond comparative analyses of surgical approaches, several experimental and clinical investigations have profoundly influenced PD. The randomized controlled trial by Figueras et al. comparing pancreaticogastrostomy versus pancreatojejunostomy demonstrated no significant difference in clinically relevant postoperative pancreatic fistula rates, suggesting that anastomotic outcomes may rely more heavily on pancreatic texture and duct size than the reconstruction technique alone [44]. Similarly, the DISPACT trial, although focused on distal pancreatectomy, was important in showcasing the technical advancements of stump closure and their relevance to pancreatic head resections [45].

Perioperative care has also advanced in the recent decades. Studies such as the PROPP trial have demonstrated the benefits of structured preoperative nutritional optimization and early postoperative enteral feeding in reducing infectious complications and enhancing recovery in major pancreatic resections [46,47]. These findings have been important in shaping ERAS pathways specifically for pancreaticoduodenectomy.

Beyond operative and perioperative advances, research in surgical oncology and translational science has developed. The integration of molecular profiling and circulating tumor DNA analysis is enabling risk stratification and treatment personalization for resectable pancreatic cancer, with implications for both surgical planning and adjuvant therapy selection [48,49]. Robotic simulation-based curricula and cadaveric models have enhanced training, with technical reproducibility in complex reconstruction, particularly for new robotic surgeons. Tam et al. demonstrated that a biotissue-based curriculum for robotic PJ significantly improved technical performance in surgical trainees, validating its role in structured robotic training pathways [50,51]. Tang et al. developed a reproducible laparoscopic PJ training model that effectively facilitated skill development in a controlled environment [52]. The predictive validity of simulation platforms has also been confirmed, with Amudhan et al. reporting strong correlations between simulator-based performance and intraoperative outcomes in pancreatic surgery [53]. The role of simulation, virtual reality, 3D printing, and hybrid models is expanding and they are becoming essential tools for advancing hepatopancreatobiliary surgical education [54]. These findings support the integration of simulation into credentialing frameworks and ongoing competency assessments, especially as minimally invasive pancreaticoduodenectomy becomes more widely adopted.

Although data remain limited, cost is a factor influencing the choice of surgical approach for PD, particularly as healthcare systems increasingly emphasize value-based care in low-resource settings. While OPD is generally considered the more economical in terms of direct intraoperative expenses, several studies have shown that LPD may reduce overall costs by shortening hospital stay and decreasing complication rates, despite longer operative times. RPD has been associated with higher intraoperative and equipment-related costs, mainly due to the robotic system, maintenance, and disposable instruments. However, in high-volume centers with optimized robotic workflows, RPD may approach cost neutrality by minimizing conversion rates, transfusions, and intensive care needs [55,56]. These findings suggest that institutional resources, surgical expertise, and long-term outcomes must be carefully weighed when evaluating the economic feasibility of minimally invasive pancreaticoduodenectomy.

Furthermore, artificial intelligence and machine learning algorithms are being explored to assist in intraoperative decision-making and postoperative risk prediction, with early applications in image-guided dissection, margin assessment, and early complication detection [57,58]. As surgical innovation accelerates, maintaining high-level evidence through well-designed prospective studies becomes crucial to inform clinical guidelines and training curricula.

An important limitation of our analysis lies in the high degree of heterogeneity observed across several outcomes, particularly for operative time, pancreatic fistula, bile leakage, and mortality. This heterogeneity likely shows the variability of clinical, methodological, and institutional factors. Differences in surgeon experience, learning curves, patient selection criteria, tumor characteristics, and perioperative management protocols all contribute to the heterogeneity in reported outcomes across studies. Additionally, the inclusion of both single- and multicenter trials and varying definitions for complications may have further amplified statistical inconsistency. The available RCTs, which are inherently difficult to standardize due to operator dependence and evolving technology platforms, also limit direct comparability. The results should be interpreted with caution, particularly for outcomes that demonstrated high inconsistency. Future studies, using standardized reporting frameworks and performed in high-volume centers, will be essential to reduce heterogeneity and strengthen the evidence.

## 5. Conclusions

This systematic review of RCTs provides a comprehensive comparative evaluation of OPD, LPD, and RPD. Our results suggest that both minimally invasive techniques offer certain advantages over the open approach, including reduced blood loss and comparable rates of postoperative morbidity and mortality. LPD was associated with significantly longer operative times but demonstrated a shorter hospital stay and lower rates of postoperative pancreatic fistula. RPD, while exhibiting higher rates of pancreatic fistula, was associated with the lowest observed mortality and similar oncologic adequacy. Notably, our analysis identified substantial heterogeneity in several outcomes, underscoring the influence of surgical experience, institutional protocols, and reporting variability. Importantly, no randomized trials have yet directly compared LPD and RPD, and only one such trial is currently registered but not yet recruiting. Until such head-to-head evidence becomes available, clinical decisions should be guided by surgeon expertise, patient factors, and institutional resources. Future multicenter RCTs are urgently needed to evaluate the relative benefits and risks of LPD versus RPD in order to establish evidence-based guidelines for the optimal surgical approach to pancreaticoduodenectomy.

## Figures and Tables

**Table 1:** Characteristics of patients and studies.

Study	Country	Total No.	OPD No.	LPD No.	RPD No.	Age (y) OPD	Age LPD	Age RPD	BMI OPD (kg/m^2^)	BMI LPD	BMI RPD	ASA I OPD/LPD/RPD	ASA II	ASA III	BD OPD (n)	BD LPD (n)	BD RPD (n)
Palanivelu et al., 2017 [15]	India	64	32	32	-	58.6	57.8	-	22.4	24.9	-	11/13/-	18/17/-	3/2/-	11	8	-
Poves et al., 2018 [16]	Spain	66	32	34	-	70	69	-	26	24	-	1/1/-	13/18/-	15/13/-	NA	NA	-
Hilst et al., 2019 [11]	Netherlands	99	49	50	-	66	67	-	26	25	-	7/5/-	26/32/-	16/13/-	26	25	-
Wang et al., 2023 [17]	China	200	100	100	-	60.7	61.9	-	22.3	22.9	-	22/32/-	54/41/-	24/27/-	37	44	-
Qin et al., 2024 [18]	China	529	261	268	-	57.8	59.2	-	22	22.4	-	44/47/-	168/158/-	49/63/-	68	63	-
Yoon et al., 2024 [19]	South Korea	235	119	116	-	63.2	62.5	-	23.6	23.9	-	6/8/-	104/102/-	17/15/-	45	55	-
Klotz et al., 2024 [21]	Germany	62	33	-	29	62.6	-	64.7	26.6	-	26.9	0/-/0	20/-/18	13/-/8	7	-	9
Liu et al., 2024 [20]	China	161	80	-	81	60.0	-	62.0	23.3	-	23.3	8/-/9	64/-/66	8/-/6	36	-	35

OPD, open pancreaticoduodenectomy; LPD, laparoscopic pancreaticoduodenectomy; RPD, robotic pancreaticoduodenectomy; BMI, body mass index; ASA, American Society of Anesthesiologists score; NA, not available.

## Data Availability

The data presented in this study are derived from previously published randomized controlled trials, all of which are publicly available through databases such as PubMed, ClinicalTrials.gov, and trial registries referenced in the manuscript. No new data were generated during this study. Full details of the included trials and extracted outcomes are available within the article.

## Abbreviations

The following abbreviations are used in this manuscript:

PD	Pancreaticoduodenectomy
OPD	Open Pancreaticoduodenectomy
LPD	Laparoscopic Pancreaticoduodenectomy
RPD	Robotic Pancreaticoduodenectomy
RCT	Randomized Controlled Trial
PRISMA	Preferred Reporting Items for Systematic Reviews and Meta-Analyses
NIH	National Institutes of Health
BMI	Body Mass Index
ASA	American Society of Anesthesiologists
CI	Confidence Interval
NMA	Network Meta-Analysis
MD	Mean Difference
SD	Standard Deviation
OR	Odds Ratio
